# Occurrence and transformation of veterinary pharmaceuticals and biocides in manure: a literature review

**DOI:** 10.1186/s12302-016-0091-8

**Published:** 2016-09-26

**Authors:** Manuel Wohde, Silvia Berkner, Thomas Junker, Sabine Konradi, Lisa Schwarz, Rolf-Alexander Düring

**Affiliations:** 1 Institute of Soil Science and Soil Conservation, IFZ, Justus Liebig University Giessen, Heinrich-Buff-Ring 26-32, 35392 Giessen, Germany; 2 Unit IV 2.2 Pharmaceuticals, Washing and Cleansing Agents and Nanomaterials, German Environment Agency (UBA), Dessau-Roßlau, Germany; 3 ECT Oekotoxikologie GmbH, Flörsheim, Germany

**Keywords:** Veterinary medicines, Drug, Pharmaceuticals, Biocides, Manure, Slurry, Transformation, Dissipation, Degradation, Monitoring

## Abstract

The spread of veterinary medicinal products (VMPs) and biocides via manure onto agriculturally used areas represents a very important emission into the environment for these product groups. Within this literature study, publicly available transformation studies with liquid manure are summarized. Transformation studies were evaluated regarding the transformation fate of tested substances, the origin and characteristics of used manure, the experimental setup, and the measured parameters. As main topics within the 42 evaluated transformation studies, the high dependency of transformation on temperature, redox potential, dry matter content, and other parameters is reported. Test duration throughout the studies ranged from 2 to 374 days and study temperature ranged from 5 to 55 °C. Only seven publications gave information on the redox potential of the manure. Further, the characterization of the matrix in many cases was inadequate due to missing parameters such as dry matter content or pH. Only three publications studied transformation of biocides. To allow for a consistent assessment of studies within the registration process, a harmonized internationally accepted and validated test method is needed. Additionally, monitoring data of VMPs in manure were collected from literature and evaluated regarding the origin and characteristics of the manure, the minimum/maximum found concentrations, and the percentage of identified compounds. Within the 27 evaluated publications, 1568 manure samples were analyzed and 39 different active substances for VMPs and 11 metabolites and transformation products of VMPs could be found in manure. Most often, the samples were analyzed for sulfonamides, tetracyclines, and fluoroquinolones. Not one study searched for biocides or worked with a non-target approach. For sulfadiazine and chlortetracycline, concentrations exceeding the predicted environmental concentrations were found.

## Background

Veterinary medicinal products (VMPs) are excreted by the treated animals in the form of unchanged parent substances and metabolized compounds. The excrements from stabled animals in Europe and North America are collected and stored mainly as liquid or solid manure before they are used as fertilizers on arable land and grassland. Biocides, which are used for the disinfection of stables, end up in the stored animal excrements. Via manure application in agriculture, veterinary medicines and biocides are released into the environment and consequently affect soil and water quality.

Depending on boundary conditions such as storage temperature, dry matter content, feeding of the animals, and availability of electron acceptors, the pharmaceuticals and biocides can be further transformed in the liquid manure. Besides transformation, other processes such as volatilization, sorption, and the formation of non-extractable residues (NER) can occur and contribute to the dissipation of the active ingredients.

Transformation products may also persist in environmental matrices and can be ecotoxic. For tetracyclines, transformation products like epimers, isomers, and anhydro-compounds were detected [5, 31]. Metabolites of sulfadiazine show transformation back to the parent compound [26]. Transformation processes are influenced by the composition of matrix, temperature, pH value, microbiology, as well as aerobic or anaerobic conditions. Compounds could adsorb to the matrix depending on its sorption capacity. The higher the dry matter content in liquid manure, the higher the number of sorption sites [35].

Generally, the transformation under aerobic conditions occurs faster than the transformation under anaerobic conditions. Also high temperatures promote the degradation of compounds in liquid manure. During manure storage in manure tanks, which is most common in Europe, the storage conditions are anaerobic. In North America, manure is often stored in lagoons because of the large amounts of manure that accumulates in large-scale concentrated animal feeding operations (CAFOs). The outdoor lagoon storage is distinguished by more aerobic conditions on the large lagoon water surface but also by anaerobic conditions in deeper layers. Composting the separated manure under aerobic conditions is a favored treatment of manure in Asia. Concluding, the transformation process of compounds is affected largely by the storage practice of manure.

There is increasing research activity regarding the transformation of single substances under laboratory conditions. Current guidance, e.g., the “guideline on determining the fate of veterinary medicinal products in manure” [19], takes transformation of VMPs and biocides in manure into account. However, there is no standardized experimental test protocol available to examine the transformation of veterinary medicinal products (VMPs) and biocides in liquid manure. The EMA guideline on transformation in manure [19] only contains basic regulatory requirements. To allow for a consistent assessment of studies within regulatory frameworks, a harmonized internationally accepted and validated test method is needed. The present review paper brings the initial situation assessment which might serve as a basis for further exploitation toward the formulation of the guideline.

This literature study first provides a survey on monitoring data of VMPs and biocides in liquid manure and secondly presents studies on transformation processes in liquid manure. The objective of this review is to consider the following questions: Which compounds are found in liquid manure? Which compounds are investigated? Which methods and analytical techniques are used and which factors have been identified affecting the transformation process in liquid manure?

## Methods

The search engines and databases such as “Google Scholar” and “ISI Web of Knowledge” were used. Categorized search items are shown in Table 1. For Boolean search, the most relevant keywords from the first and the second category were combined with one of the keywords of categories 3–6.

**Table 1:** List of categorized keywords

1	2	3	4	5	6
Manure	Transformation	Veterinary	Medicine	Biocide	Tetracycline
Slurry	Metabolism		Drug	Pesticide	Sulfonamide
Feces	Catabolism		Pharmaceutical	Disinfectant	Antibiotic
Faeces	Anabolism				Antiparasitic
Lagoon	Degradation				
	Decomposition				
	Dissipation				
	Fate				
	Reaction				
	Conversion				
	Management				

International publications from the year 2000 to date were considered. In addition, cross-references of the found publications were evaluated. Further, relevant German authorities and organizations (German Federal State Ministries and Departments, private associations) were asked for reports on related topics.

By this means, 668 individual publications were obtained, 27 of which were found to measure or monitor the occurrence of VMPs in liquid manure. Examined substances, determined concentrations, origin of the manure, and further parameters were listed. From 668 records, 42 publications deal explicitly with transformation of veterinary medicinal products and biocides in manure. These citations were evaluated systematically, considering specific parameters such as investigated compounds and substance amounts, characterization of matrices, transformation products, methodology, and chemical analysis of the studies. Liquid manure generally is defined in the following way: *“Liquid manure that is the predominant type of manure in Europe and North America is a mixture of urine, faces and water used to clean the stables and may also contain bedding material. Typical dry matter contents for pig and cattle manure have been found to be 5 and 10* *%, respectively”* [74]. Since many studies do not report the dry matter content of the analyzed manure or do not follow the common definition of liquid manure, only those transformation studies were excluded from this literature review, that clearly work with stable manure from manure heaps.

## Occurrence of veterinary medicines and biocides in manure

The monitoring data tables (Tables 2, 3, 4, 5) summarize the results of 27 different publications measuring active ingredients of veterinary medicinal products in manure from the year 2000 until today. The analyses were conducted in North America (Canada), in Europe (Austria, Czech Republic, Denmark, Germany, Italy, Switzerland), and in Asia (China, Japan). Although the results give only information on specific locations, the ubiquitous occurrence of active ingredients in manure is demonstrated. It can be assumed that whenever veterinary medicinal products are used, portions of these will be found in the manure.

**Table 2:** Sulfonamides and their metabolites and transformation products found in manure

Substance	Reference	Matrix	Origin	Min	Max	Unit	Dry matter content, comments or quotation	*n*	*n* positive	% positive
Sulfachloropyridazine	[28]	Pig and poultry manure	China	0.340	3.660	mg/kg (dw)	“Liquid swine manure” (ns)	6	2	33
	[79]	Pig, cattle and poultry manure	China	0.090	3.510	mg/kg (dw)	(ns)	143	7	5
Sulfadiazine	[20]	Pig manure	Germany (NI)	0.700	235.100	mg/kg (ww)	0.5–16.8 % (mean 5 %)	344	100	29
	[23]	Pig manure	Germany	3.500	11.300	mg/kg (dw)	9.6–9.8 %	3	2	67
	[25]	Pig manure	Germany (BY)	0.100	5.000	mg/kg (ww)	0.2–17.3 % (mean 3.7 %)	380	19	5
	[28]	Pig and poultry manure	China	0.160	0.780	mg/kg (dw)	“Liquid swine manure” (ns)	6	2	33
	[31]	Pig manure	Denmark	0.630	2.100	mg/kg (dw)	2.8–13.4 %	6	2	33
	[55]	(ns)	Germany	0.011	0.080	mg/kg (ns)	“Liquid manure” (ns)	4	2	50
	[56]	Pig and cattle manure	Germany (NW)	–	0.650	mg/kg (dw)	Liquid and stable manure (ns)	34	5	15
	[77]	Pig manure	Germany	0.700	35.300	mg/kg (ww)	0.7–16.11 %	176	86	49
	[79]	Pig, cattle and poultry manure	China	0.020	3.120	mg/kg (dw)	(ns)	143	14	10
4-Hydroxy-sulfadiazine	[56]	Pig and cattle manure	Germany (NW)	–	9.050	mg/kg (dw)	Liquid and stable manure (ns)	34	8	24
*N*4-Acetyl-sulfadiazine	[25]	Pig manure	Germany (BY)	–	–	not quantified	0.2–17.3 % (mean 3.7 %)	380	19	5
	[55]	(ns)	Germany	0.010	0.270	mg/kg (ns)	“Liquid manure” (ns)	4	2	50
	[56]	Pig and cattle manure	Germany (NW)	–	0.150	mg/kg (dw)	Liquid and stable manure (ns)	34	6	18
Sulfadimethoxine	[25]	Pig manure	Germany (BY)	0.050	0.600	mg/kg (ww)	0.2–17.3 % (mean 3.7 %)	380	5	1
	[54]	Pig manure	China	0.120	1.255	mg/kg (dw)	(ns)	126	3	2
Sulfadimidine	[48]	Pig manure	Austria	–	<20	mg/kg (dw)	“Liquid manure” (ns)	30	18	60
	[8]	Cattle manure	Canada	–	9.990	mg/kg (dw)	24.4–44.4 % (mean 37 %)	6	4	67
	[13]	Pig manure	Switzerland	–	14.400	mg/L (ww)	“In the supernatant” (water phase) (ns)	1	1	100
	[15]	Pig manure	Germany	1.000	1.100	mg/kg (ww)	(ns)	2	2	100
	[15]	Cattle manure	Germany	<0.1	<0.1	mg/kg (ww)	(ns)	2	2	100
	[20]	Pig manure	Germany (NI)	0.700	167.000	mg/kg (ww)	0.5–16.8 % (mean 5 %)	344	183	53
	[23]	Pig manure	Germany	–	7.200	mg/kg (dw)	9.6–9.8 %	3	1	33
	[54]	Pig manure	China	0.011	28.700	mg/kg (dw)	(ns)	126	65	52
	[56]	Pig and cattle manure	Germany (NW)	–	7.040	mg/kg (dw)	Liquid and stable manure (ns)	34	6	18
	[58]	Pig manure	Germany	0.130	20.000	mg/kg (dw)	1.2–28 %	30	18	60
	[75]	Pig manure	Germany (BY)	0.140	1.700	mg/L (ww)	1–2 %	8	8	100
	[77]	Pig manure	Germany	0.700	167.000	mg/kg (ww)	0.7–16.13 %	176	85	48
	[79]	Pig, cattle and poultry manure	China	0.060	6.040	mg/kg (dw)	(ns)	143	17	12
	[22]	Pig and cattle manure	Switzerland	0.130	8.700	mg/kg (ww)	1.1–3.7 %	6	6	100
	[25]	Pig manure	Germany (BY)	0.050	38.000	mg/kg (ww)	0.2–17.3 % (mean 3.7 %)	380	181	48
	[55]	(ns)	Germany	0.011	0.062	mg/kg (ns)	“Liquid manure” (ns)	4	2	50
*N*4-Acetyl-sulfadimidine	[22]	Pig and cattle manure	Switzerland	<0.1	2.600	mg/kg (ww)	1.1–3.7 %	6	5	83
	[25]	Pig manure	Germany (BY)	0.050	27.000	mg/kg (ww)	0.2–17.3 % (mean 3.7 %)	380	117	31
	[75]	Pig manure	Germany (BY)	0.120	1.000	mg/L (ww)	1–2 %	8	8	100
Sulfadoxine	[28]	Pig and poultry manure	China	0.350	0.710	mg/kg (dw)	“Liquid swine manure” (ns)	6	3	50
	[31]	Pig manure	Denmark	0.015	0.220	mg/kg (dw)	2.8–13.4 %	6	3	50
Sulfaguanidine	[79]	Pig, cattle and poultry manure	China	0.010	1.550	mg/kg (dw)	(ns)	143	27	19
Sulfamerazine	[25]	Pig manure	Germany (BY)	0.700	0.900	mg/kg (ww)	0.2–17.3 % (mean 3.7 %)	380	7	2
	[79]	Pig, cattle and poultry manure	China	0.090	0.660	mg/kg (dw)	(ns)	143	6	4
*N*4-Acetyl-Sulfamerazine	[25]	Pig manure	Germany (BY)	–	–	not quantified	0.2–17.3 % (mean 3.7 %)	380	5	1
Sulfamethizole	[54]	Pig manure	China	0.052	2.422	mg/kg (dw)	(ns)	126	35	28
Sulfamethoxazole	[25]	Pig manure	Germany (BY)	0.050	0.050	mg/kg (ww)	0.2–17.3 % (mean 3.7 %)	380	3	1
	[28]	Pig and poultry manure	China	0.340	1.290	mg/kg (dw)	“Liquid swine manure” (ns)	6	2	33
	[51]	Pig manure	Japan	0.002	0.035	mg/kg (ns)	(ns)	5	4	80
	[51]	Cattle manure after fermentation	Japan	–	0.010	mg/kg (ns)	(ns)	8	1	13
	[54]	Pig manure	China	0.137	0.639	mg/kg (dw)	(ns)	126	6	5
	[58]	Pig manure	Germany	<0.1	2.400	mg/kg (dw)	1.2–28 %	30	2	7
	[79]	Pig, cattle and poultry manure	China	0.120	2.800	mg/kg (dw)	(ns)	143	7	5
Sulfamethoxypyridazine	[56]	Pig and cattle manure	Germany (NW)	–	0.020	mg/kg (dw)	Liquid and stable manure (ns)	34	4	12
Sulfamonomethoxine	[51]	Pig manure	Japan	–	0.210	mg/kg (ns)	(ns)	5	1	20
	[51]	Cattle manure after fermentation	Japan	–	0.022	mg/kg (ns)	(ns)	8	1	13
	[79]	Pig, cattle and poultry manure	China	0.060	4.080	mg/kg (dw)	(ns)	143	39	27
Sulfanilamide	[79]	Pig, cattle and poultry manure	China	0.020	1.590	mg/kg (dw)	(ns)	143	5	3
Sulfaquinoxaline	[56]	Pig and cattle manure	Germany (NW)	–	0.670	mg/kg (dw)	Liquid and stable manure (ns)	34	3	9
Sulfathiazole	[22]	Pig and cattle manure	Switzerland	0.100	12.400	mg/kg (ww)	1.1–3.7 %	6	4	67
	[25]	Pig manure	Germany (BY)	0.050	0.100	mg/kg (ww)	0.2–17.3 % (mean 3.7 %)	380	5	1
	[54]	Pig manure	China	0.312		mg/kg (dw)	(ns)	126	1	1

*dw* dry weight, *ww* wet weight, *ns* not specified

**Table 3:** Tetracyclines and their metabolites and transformation products found in manure

Substance	Reference	Matrix	Origin	Min	Max	Unit	Dry matter content, comments or quotation	*n*	*n* positive	% positive
Chlortetracycline	[48]	Pig manure	Austria	0.100	46.000	mg/kg (dw)	“Liquid manure” (ns)	30	17	57
	[20]	Pig manure	Germany (NI)	1.100	330.700	mg/kg (ww)	0.5–16.8 % (mean 5 %)	344	44	13
	[24]	Pig manure	Germany	0.090	0.100	mg/kg (ww)	(ns)	2	2	100
	[23]	Pig manure	Germany	0.900	1.000	mg/kg (dw)	9.6–9.8 %	3	2	67
	[25]	Pig manure	Germany (BY)	0.100	50.800	mg/kg (ww)	0.2–17.3 % (mean 3.7 %)	380	140	37
	[28]	Pig and poultry manure	China	0.150	14.700	mg/kg (dw)	“Liquid swine manure” (ns)	6	4	67
	[31]	Pig manure	Denmark	1.100	15.700	mg/kg (dw)	2.8–13.4 %	6	5	83
	[51]	Pig manure	Japan	0.240	0.280	mg/kg (ns)	(ns)	5	2	40
	[51]	Cattle manure after fermentation	Japan	–	0.001	mg/kg (ns)	(ns)	8	1	13
	[54]	Pig manure	China	0.053	764.407	mg/kg (dw)	(ns)	126	122	97
	[56]	Pig and cattle manure	Germany (NW)	–	3.600	mg/kg (dw)	Liquid and stable manure (ns)	34	7	21
	[58]	Pig manure	Germany	0.100	46.000	mg/kg (dw)	1.2–28 %	30	17	57
	[66]	Pig manure	Czech Republic	–	5.880	mg/kg (ns)	“Liquid hog manure” (ns)	5	1	20
	[75]	Pig manure	Germany (BY)	0.600	2.000	mg/L (ww)	1–2 %	3	3	100
	[77]	Pig manure	Germany	1.100	25.700	mg/kg (ww)	0.7–16.1 %	176	18	10
	[79]	Pig, cattle and poultry manure	China	0.160	27.590	mg/kg (dw)	(ns)	143	72	50
Epi-chlortetracycline	[31]	Pig manure	Denmark	1.700	14.100	mg/kg (dw)	2.8–13.4 %	6	5	83
Doxycycline	[25]	Pig manure	Germany (BY)	0.100	0.700	mg/kg (ww)	0.2–17.3 % (mean 3.7 %)	380	5	1
	[31]	Pig manure	Denmark	0.550	3.100	mg/kg (dw)	2.8–13.4 %	6	6	100
	[66]	Pig manure	Czech Republic	–	0.990	mg/kg (ns)	“Liquid hog manure” (ns)	5	1	20
	[79]	Pig, cattle and poultry manure	China	0.230	13.500	mg/kg (dw)	(ns)	143	21	15
Metacycline	[79]	Pig, cattle and poultry manure	China	0.140	5.860	mg/kg (dw)	(ns)	143	50	35
Oxytetracycline	[48]	Pig manure	Austria	0.290	29.000	mg/kg (dw)	“Liquid manure” (ns)	30	22	73
	[43]	Cattle manure	Italy	–	19.000	mg/kg (ns)	“Heap” (ns)	1	1	100
	[20]	Pig manure	Germany (NI)	1.600	136.200	mg/kg (ww)	0.5–16.8 % (mean 5 %)	344	10	3
	[25]	Pig manure	Germany (BY)	0.100	0.900	mg/kg (ww)	0.2–17.3 % (mean 3.7 %)	380	16	4
	[31]	Pig manure	Denmark	0.048	1.500	mg/kg (dw)	2.8–13.4 %	6	3	50
	[51]	Pig manure	Japan	–	0.013	mg/kg (ns)	(ns)	5	1	20
	[51]	Cattle manure after fermentation	Japan	–	0.001	mg/kg (ns)	(ns)	8	1	13
	[54]	Pig manure	China	0.044	172.874	mg/kg (dw)	(ns)	126	114	90
	[56]	Pig and cattle manure	Germany (NW)	–	1.490	mg/kg (dw)	Liquid and stable manure (ns)	34	5	15
	[58]	Pig manure	Germany	0.210	29.000	mg/kg (dw)	1.2–28 %	30	22	73
	[77]	Pig manure	Germany	1.600	136.200	mg/kg (ww)	0.7–16.9 %	176	9	5
	[79]	Pig, cattle and poultry manure	China	0.150	59.590	mg/kg (dw)	(ns)	143	50	35
	[33]	Cattle manure	Turkey	–	0.060	mg/kg (ns)	(ns)	1	1	100
Epi-oxytetracycline	[31]	Pig manure	Denmark	0.330	0.450	mg/kg (dw)	2.8–13.4 %	6	2	33
Tetracycline	[48]	Pig manure	Austria	0.360	23.000	mg/kg (dw)	“Liquid manure” (ns)	30	22	73
	[24]	Pig manure	Germany	3.200	4.000	mg/kg (ww)	(ns)	2	2	100
	[23]	Pig manure	Germany	14.100	41.200	mg/kg (dw)	9.6–9.8 %	3	3	100
	[25]	Pig manure	Germany (BY)	0.100	46.000	mg/kg (ww)	0.2–17.3 % (mean 3.7 %)	380	111	29
	[28]	Pig and poultry manure	China	0.180	0.840	mg/kg (dw)	“Liquid swine manure” (ns)	6	4	67
	[31]	Pig manure	Denmark	0.091	1.600	mg/kg (dw)	2.8–13.4 %	6	5	83
	[51]	Pig manure	Japan	0.005	0.015	mg/kg (ns)	(ns)	5	3	60
	[51]	Cattle manure after fermentation	Japan	–	0.001	mg/kg (ns)	(ns)	8	2	25
	[54]	Pig manure	China	0.037	19.417	mg/kg (dw)	(ns)	126	107	85
	[56]	Pig and cattle manure	Germany (NW)	–	2.450	mg/kg (dw)	Liquid and stable manure (ns)	34	12	35
	[58]	Pig manure	Germany	0.360	23.000	mg/kg (dw)	1.2–28 %	30	22	73
	[78]	Pig manure	Germany (NW)	0.600	66.000	mg/L (ww)	“Pig slurry” (ns)	181	43	24
	[77]	Pig manure	Germany	0.900	43.100	mg/kg (ww)	0.7–16.8 %	176	87	49
	[20]	Pig manure	Germany (NI)	0.700	45.700	mg/kg (ww)	0.5–16.8 % (mean 5 %)	344	152	44
Epi-tetracycline	[31]	Pig manure	Denmark	0.061	0.990	mg/kg (dw)	2.8–13.4 %	6	5	83

*dw* dry weight, *ww* wet weight, *ns* not specified

**Table 4:** Fluorchinolones found in manure

Substance	Reference	Matrix	Origin	Min	Max	Unit	Dry matter content, comments or quotation	*n*	*n* positive	% positive
Ciprofloxacin	[51]	Pig manure	Japan	–	0.006	mg/kg (ns)	(ns)	5	1	20
	[51]	Cattle manure after fermentation	Japan	0.002	0.012	mg/kg (ns)	(ns)	8	4	50
	[56]	Pig and cattle manure	Germany (NW)	–	0.070	mg/kg (dw)	Liquid and stable manure (ns)	34	3	9
	[58]	Pig manure	Germany	0.180	0.620	mg/kg (dw)	1.2–28 %	30	4	13
	[75]	Pig manure	Germany (BY)	0.005	0.028	mg/L (ww)	1–2 %	5	5	100
	[79]	Pig, cattle and poultry manure	China	0.490	45.590	mg/kg (dw)	(ns)	143	44	31
Danofloxacin	[56]	Pig and cattle manure	Germany (NW)		0.050	mg/kg (dw)	Liquid and stable manure (ns)	34	1	3
	[79]	Pig, cattle and poultry manure	China	0.080	3.060	mg/kg (dw)	(ns)	143	39	27
Difloxacin	[79]	Pig, cattle and poultry manure	China	0.410	12.380	mg/kg (dw)	(ns)	143	8	6
Enrofloxacin	[48]	Pig manure	Austria	0.130	0.750	mg/kg (dw)	“Liquid manure” (ns)	–	–	–
	[56]	Pig and cattle manure	Germany (NW)		0.550	mg/kg (dw)	Liquid and stable manure (ns)	34	5	15
	[58]	Pig manure	Germany	0.130	0.750	mg/kg (dw)	1.2–28 %	30	5	17
	[75]	Pig manure	Germany (BY)	0.050	0.116	mg/L (ww)	1–2 %	5	5	100
	[79]	Pig, cattle and poultry manure	China	0.330	1420.760	mg/kg (dw)	(ns)	143	67	47
Fleroxacin	[79]	Pig, cattle and poultry manure	China	0.760	99.430	mg/kg (dw)	(ns)	143	35	24
Levofloxacin	[51]	Pig manure	Japan	–	0.003	mg/kg (ns)	(ns)	5	1	20
	[51]	Cattle manure after fermentation	Japan	0.001	0.002	mg/kg (ns)	(ns)	8	2	25
Lomefloxacin	[79]	Pig, cattle and poultry manure	China	0.610	44.160	mg/kg (dw)	(ns)	143	45	31
Marbofloxacin	[56]	Pig and cattle manure	Germany (NW)	–	0.050	mg/kg (dw)	Liquid and stable manure (ns)	34	3	9
Norfloxacin	[79]	Pig, cattle and poultry manure	China	0.560	225.450	mg/kg (dw)	(ns)	143	37	26
Ofloxacin	[28]	Pig and poultry manure	China	0.450	3.870	mg/kg (dw)	“Liquid swine manure” (ns)	6	2	33
Orbifloxacin	[56]	Pig and cattle manure	Germany (NW)	–	0.020	mg/kg (dw)	Liquid and stable manure (ns)	34	1	3
Sarafloxacin	[56]	Pig and cattle manure	Germany (NW)	–	0.060	mg/kg (dw)	Liquid and stable manure (ns)	34	1	3

*dw* dry weight, *ww* wet weight, *ns* not specified

**Table 5:** Other veterinary medicines and its metabolites and transformation products found in manure

Substance	Reference	Matrix	Origin	Min	Max	Unit	Dry matter content, comments or quotation	*n*	*n* positive	% positive
Flubendazole	[75]	Pig manure	Germany (BY)	0.020	0.056	mg/L (ww)	1–2 %	7	7	100
Amino-flubendazole	[75]	Pig manure	Germany (BY)	0.032	0.110	mg/L (ww)	1–2 %	7	7	100
Hydroxy-flubendazole	[75]	Pig manure	Germany (BY)	0.018	0.075	mg/L (ww)	1–2 %	7	7	100
Lincomycin	[39]	Pig manure	Canada	2.520	9.780	mg/L (ww)	mean 2.4 %	5	5	100
Salinomycin	[60]	Pig manure	Germany	–	0.011	mg/kg (ns)	5 %	4	1	25
Spectinomycin	[39]	Pig manure	Canada	0.173	0.686	mg/L (ww)	mean 2.4 %	5	5	100
Tiamulin	[25]	Pig manure	Germany (BY)	–	0.500	mg/kg (ww)	0.2–17.3 % (mean 3.7 %)	380	1	<1
	[54]	Pig manure	China	0.076	0.169	mg/kg (dw)	(ns)	126	6	5
	[60]	Pig manure	Germany	–	0.043	mg/kg (ns)	5 %	4	1	25
Toltrazuril	[53]	Pig manure	Denmark	–	0.114	mg/kg (dw)	“Manure from a slurry storage tank” (ns)	1	1	100
Toltrazuril sulfone	[53]	Pig manure	Denmark	–	0.085	mg/kg (dw)	“Manure from a slurry storage tank” (ns)	1	1	100
Toltrazuril sulfoxide	[53]	Pig manure	Denmark	–	0.007	mg/kg (dw)	“Manure from a slurry storage tank” (ns)	1	1	100
Trimethoprim	[22]	Pig and cattle manure	Switzerland	<0.1	<0.1	mg/kg (ww)	1.1–3.7 %	6	1	17
	[56]	Pig and cattle manure	Germany (NW)	–	0.050	mg/kg (dw)	Liquid and stable manure (ns)	34	1	3
Tylosin	[43]	Cattle manure	Italy	–	<0.25	mg/kg (ns)	“Heap” (ns)	1	1	100
	[63]	Pig manure	Canada	0.030	0.543	mg/kg (dw)	(ns)	–	–	–
	[75]	Pig manure	Germany (BY)	0.130	0.320	mg/L (ww)	1–2 %	8	8	100

*dw* dry weight, *ww* wet weight, *ns* not specified

In some studies, a lot of samples were taken covering a large number of different manures—up to 380 samples in Harms [25]—and in other studies only individual manures were sampled after medication. In 19 studies, only pig manure was analyzed, whereas three studies worked with cattle manure and four studies worked with pig, cattle, or poultry manure. One publication does not specify the origin of the analyzed manure.

Manure and liquid manure samples with different dry matter contents are considered in this literature study (range 0.2–44.4 %). Sixteen of 27 studies do not specify dry matter content as a basic parameter. Twelve of 27 studies report concentrations of substances in manure in mg/kg dry weight (dw), 10 of 27 studies work with mg/kg wet weight (ww), and five studies do not specify (ns) whether they calculated concentrations on the basis of dry or wet weight. Because of this, it is difficult to compare the found concentrations of the single active substances. The lowest values are found at the µg/kg order of magnitude—often restricted by the limit of detection (LOD) of the analytical method.

Among all the reviewed literature, 39 different active substances of VMPs were found in manure. Moreover, 11 metabolites and transformation products of active substances were identified. For this, 1568 manure samples were analyzed within the 27 publications. Mainly, the samples were analyzed for sulfonamides, tetracyclines, and fluoroquinolones. None of the studies worked with a non-target approach or searched for biocides. By far, the most frequently found single active substances are sulfadimidine (599 positive), tetracycline (575 positive), and chlortetracycline (457 positive). There are six publications each of which analyzed more than 100 manure samples. All of these are from Chinese or German institutes. The active substances with the highest percentage of positive findings (>50 %) within these six publications are chlortetracycline, oxytetracycline, tetracycline, and sulfadimidine.

The 15 highest concentrations were found in pig manure from Germany or China. The highest concentration was 1420.76 mg/kg (dw) of enrofloxacin, found in poultry manure from China, followed by 764.407 mg/kg (dw) chlortetracycline in pig manure from China and 330.7 mg/kg (ww) in pig manure from Germany. Further, very high values were found for other sulfonamides, tetracyclines, and fluorchinolones.

More recently, Berendsen et al. [10] developed a comprehensive method for the analysis of 44 antibiotic compounds in animal feces by liquid chromatography coupled with tandem mass spectrometry (LC–MS/MS). As the study analyzed animal feces and not liquid manure, this study was not taken into account for Tables 2–5, although the measured VMPs will obviously end up in liquid manure. In 34 % of the samples, more than one antibiotic was detected.

### Predicted environmental concentrations vs. measured environmental concentrations

With the summarized measured environmental concentrations (MECs) in Tables 2–5, it is possible to compare them with predicted environmental concentrations (PECs) in liquid manure as they are calculated for an environmental risk assessment of VMPs according to the EMA guidance [18]. With this analysis, the concept of PECs can be evaluated, since MECs are not relevant for the environmental risk assessment of VMPs. The guideline works with concentrations relating to the wet weight (mg/kg) and the nitrogen content of the manure, so that PECs in manure are given by

$${\text{PEC}}_{\text{ww manure}} = \frac{{D \times {\text{Ad}} \times {\text{Bw}} \times {\text{Fh}}}}{\text{Ns}} \times \frac{{N_{\text{total}} }}{1000 }$$

with PEC_ww manure_ being the predicted environmental concentration in manure (mg/kg_wet manure_), *D* the daily dose of the active ingredient (mg/kg_bw_ day), Ad the number of days of treatment (day), Bw the animal body weight (kg_bw_), Fh the fraction of herd treated (value between 0 and 1), Ns the nitrogen produced during storage time (kg N), and *N*_total_ the nitrogen content of the specific manure (kg N/kg_wet manure_).

The default values for Bw, Fh, and Ns are given within the EMA guideline [18], with respect to the type of animal (calf, dairy cow, cattle 0–1 year or >2 years, weaner pig, fattening pig, sow). Unfortunately, the exact animal type cannot often be deduced from the 27 publications, so that the simplified min./max. values of the term Bw/Ns of the whole species cattle or pig were used for the calculation of min./max. PEC_ww manure_ values. For cattle, this results in minimum and maximum values of Bw/Ns of 28.33 and 56 kg_bw_/(kg N), respectively. For pigs, these values lie between 34.21 and 37.88 kg_bw_/(kg N).

The recommended daily doses (*D*) and the number of days of animal treatment (Ad) for specific products are given under point 4.9, in the summary of product characteristics (SPC). These parameters are, e.g., accessible via the product databases of the Veterinary Medicines Directorate of the United Kingdom [71] or via the drug information portal of the German Federal Ministry of Health [12]. All registered VMPs containing the frequently found active ingredients such as chlortetracycline, oxytetracycline, tetracycline, sulfadimidine, and sulfadiazine were chosen for consideration of PEC_ww manure_ values (oral or subcutaneous administration). For this, the minimum and maximum values of *D* × Ad were used for min./max. calculation of PEC_ww manure_ (Table 6). The fraction of herd treated (Fh) was set to 1 [18].

**Table 6:** Predicted environmental concentrations and measured environmental concentrations in manure as given in Tables 2–5 (PEC_ww manure_ and MEC_ww manure_) of the five most frequently monitored and found VMPs in (liquid) manure

Active ingredient (number of registered products considered)	Species	Min.–max. PEC_ww manure_ (mg/kg_ww_)	Highest MECs_ww manure_ (mg/kg_ww_)
Chlortetracycline (19)	Cattle	10.9–24.9	–
	Pig	4.0–154.7	*330.7, 764.4*
Oxytetracycline (30)	Cattle	0.49–44.5	**–**
	Pig	0.48–182	136.2
Tetracycline (8)	Cattle	10.9–72.8	–
	Pig	31.7–216.6	66.0
Sulfadimidine (19)	Cattle	4.4–124.6	–
	Pig	3.2–254.8	167.0
Sulfadiazine (21)	Cattle	1.6–22.3	–
	Pig	1.0–45.5	*235.1*

Among the MECs_ww manure_, only those for which the concentrations in liquid manure were clearly given as mg/kg_wet weight_ were considered

MECs_ww manure_ exceeding the max. PEC_ww manure_ are italicized

Unfortunately, only very few of the monitoring studies report the nitrogen content of the analyzed manures. Additionally, the exact animal type is also very often not given within the 27 publications, as mentioned before. For this, minimum and maximum nitrogen contents of the different liquid manure types (*N*_total_) were taken from secondary literature. Cattle liquid manure has a total nitrogen content between 3.2 and 4.7 g/L, whereas the nitrogen content of pig liquid manure ranges between 2.8 and 6.5 g/L [46]. For dry matter content below 25 %, a density of 1 g/mL can be assumed for liquid manure [7] so that nitrogen content can also be given as g/kg. PEC_dw manure_ values have not been calculated (dw: dry weight), as theoretical dry matter content of liquid manure is subject to a further high variability. Although the concentrations related to the dry weight of manure are generally more reliable than those given as wet weight concentrations.

The estimated PECs_ww manure_ are based on the total residue approach, i.e., metabolism of the VMPs was not taken into account. Moreover, it is concerning that for liquid manure from pigs, two publications reveal MECs of chlortetracycline, exceeding the highly conservative maximum PEC_ww manure_ by a factor of two and five. Several further publications report MECs_ww manure_ for chlortetracycline, which exceed the minimum PEC_ww manure_. Also for sulfadiazine one MEC_ww manure_ exceeded the max. PEC_ww manure_. For sulfadimidine, tetracycline, and oxytetracycline, MECs are in the same order of magnitude as the calculated PEC_ww manure_. Considering injection products, the PEC_ww manure_ is also exceeded by the highest MEC for oxytetracycline in Table 3.

## Transformation of VMPs and biocides in liquid manure

The focus of this literature research was on transformation studies using liquid manure and manure from lagoons. Liquid manure is the substrate found in manure tanks, which consists of urine, feces, and sometimes bedding material and water from cleaning the stables. It is important to note the difference to dung or excrements, which are distinguished from manure by being directly excreted and not collected and stored for longer time periods during which anaerobic conditions develop [74]. In this review, also some studies using excrements and related matrices were included in order to get a comprehensive picture of available methods.

To study the environmental fate of VMPs, many different studies can be found, using mixtures of soil and manure or test systems containing additional plants. These are not considered for the survey. Studies on solid manure (mainly conducted at Asian institutions) are also not considered in this review as the composition of this material is considerably more variable than the composition of liquid manure, which results in e.g., wide ranges of oxygen availability. Compared to solid manure, liquid manure exhibits a more homogeneous composition. This type of manure was considered primarily, as it has been found to be the predominant type of manure in the EU countries and North America [74]. Generally, the research on the transformation of pharmaceuticals in manure focuses on North America, Europe, and Asia. There are some studies working on manure treatment technologies and some studies working on the effects of VMPs on biogas production without studying transformation. Those studies are also not considered here.

A limited number of 42 relevant studies dealing with the transformation of VMPs and biocides in liquid manure could be found. These studies are assorted in Tables 7 and 8, together with information on their experimental design. On the whole, there are only scarce data on the transformation of veterinary medicinal products. Especially on the transformation of biocides only three publications were found. However, there is an increasing publication rate worldwide, which reflects the interest in and relevance of this research field.

**Table 7:** Studies on the transformation of VMPs and biocides in liquid manure and similar matrices (excrements, biosolids, etc., as specified in the second last column)

Author (Year)	Substances	Substance class	TP	Initial concentration	DT_50_	Mineralization	Manure (type and source)	Dry matter
Akyol et al. (2016) [1]	Oxytetracycline	Tetracycline	+	1.51–2.57 mg/L	13–17 days	nd	Cattle (medicated, mixed excrements)	4.0, 5.5 %
Ali et al. (2013) [2]	Tylosin	Macrolide	−	160 mg/L	nd (highly pH and Eh dependant)	nd	Cattle (spiked, mixed lagoon sediment)	2.7 %
Álvarez et al. (2010) [3]	Oxytetracycline (OTC), Chlortetracycline (CTC)	Tetracycline	+	10, 50, 100 mg/L	15.4–12.0 (OTC), 4.1–3.2 (CTC) days	nd	Pig (spiked, tank)	nd
Angenent et al. (2008) [4]	Tylosin A	Macrolide	+	5.8 mg/L (measured)	2.49 h	nd	Pig (spiked, tank/ASBR)	nd
Arikan (2008) [5]	Chlortetracycline	Tetracycline	+	1.0 and 5.9 mg/L (buffer extraction, pH 4)	18 days	nd	Pig (medicated, mixed excrements)	5 %
Arikan et al. (2006) [6]	Oxytetracycline	Tetracycline	+	9.8 mg/L	56 days	nd	Cattle (medicated, mixed excrements)	5 %
Bailey et al. (2016) [9]	Sulfadiazine, sulfadimidine, sulfamethoxazole, tetracycline	Sulfonamide, tetracycline	−	Each 10 mg/L	nd	nd	Cattle (medicated, mixed excrements)	5, 10, 15 %
Blackwell et al. (2005) [11]	Oxytetracycline (OTC), sulfachloropyridazine (SCP)	Tetracycline, sulfonamide	−	19.2 (OTC), 26.1 (SCP) mg/L	79 (OTC), 127 (SCP) days	nd	Pig (spiked, tank)	2 %
Cetecioglu et al. (2013) [14]	Tetracycline	Tetracycline	−	Gradient: 1.65, 5.7, 8.5 mg/L	nd	nd	Synthetic (spiked, ASBR)	nd
Grote et al. (2004) [21]	Chlortetracycline (CTC), sulfadiazine (SDZ), trimethoprim (TMP)	Tetracycline, sulfonamide	+	Up to: 87.5 (CTC), 498.9 (SDZ), 15.8 (TMP) mg/kg	nd	nd	Pig (medicated, “barrels”)	nd
Harms (2006) [25]	20 different substances	Tetracycline, sulfonamide, and others	−	Numerous, many not given	nd	nd	Pig (medicated and spiked, tank)	nd
Heuer et al. (2008) [26]	Sulfadiazine (^14^C)	Sulfonamide	+	>80 mg/kg	nd (DT_50_ not reached)	<1 %	Pig (medicated, mixed excrements)	6 %
Höltge and Kreuzig (2007) [27]	Sulfamethoxazole, acetyl-sulfamethoxazole (each ^14^C)	Sulfonamide and metabolite	+	3 mg/kg	nd	≤1 %	Cattle (spiked, mixed excrements)	13 %
Huang et al. (2014) [29]	Chlortetracycline	Tetracycline	−	0.55 mg/g	nd	nd	Pig (medicated, spiked, mixed excrements)	nd
Joy et al. (2014) [32]	Bacitracin (BAC), chlortetracycline (CTC), tylosin (TYL)	Polypeptide antibiotic, tetracycline, macrolide	+	50 (BAC), 300 (CTC), 10 (TYL) mg/kg	1.9 (BAC), 1 (CTC), 9.7 (TYL) d	nd	Pig (medicated, mixed excrements)	0.84(BAC), 0.37(CTC), 0.89(TYL) %
Kolz et al. (2005) [34]	Tylosin	Macrolide	+	20 and 195 mg/L	DT90: 40–500 h	nd	Pig (spiked, lagoon water)	1.5, 3.6 %
Kreuzig (2010) [35]	Erythromycin (ERY), sulfamethoxazole (SMZ), cyanamide^a^ (CYN), imazalil^a^ (IMZ), (each ^14^C)	Macrolide, sulfonamide, biocide, imidazole	−	Only absolute radioactivity given; 0.1–0.2 MBq	nd	<0.1 % (ERY, SMZ); 28 % (CYN); nd for (IMZ)	Pig, cattle (spiked, mixed excrements)	2.5, 5, 10 %
Kreuzig and Höltge (2005) [38]	Sulfadiazine (^14^C)	Sulfonamide	−	500 µg/kg	17 days	1 %	Cattle (spiked, mixed excrements)	13 %
Kreuzig et al. (2007) [36]	Fenbendazole (FEN), flubendazole (FLU), (each ^14^C)	Benzimidazole	+	200 (FEN), 2500 (FLU) µg/kg	nd (DT_50_ not reached)	<0.6 %	Pig (spiked, mixed excrements)	3–13 %
Kreuzig et al. (2010) [37]	Imazalil^a^ (^14^C)	Imidazole	+	4.3 and 4.5 mg/kg	>177 days	0.1 %	Pig, cattle (spiked, mixed excrements)	2.5, 5, 10 %
Kuchta and Cessna (2009) [39]	Lincomycin (LIN), spectinomycin (SPN)	Antimicrobial	−	38.7 (LIN), 387 (SPN) µg/L	nd	nd	Pig (spiked, lagoon water)	nd
Kühne et al. (2000) [40]	Tetracycline	Tetracycline	+	200 mg/L	9 days	nd	Pig (spiked, tank)	nd
Lamshöft et al. (2010) [41]	Difloxacin (DIF), sulfadiazine (SDZ), (each ^14^C)	Fluoroquinolone, sulfonamide	+	17.1 ± 0.4 (DIF), 156.0 ± 4.2 (SDZ) mg/L	nd (DT_50_ not reached)	0.2 % (DIF), 0.5 % (SDZ)	Pig (medicated, mixed excrements)	3.3–6 %
Li et al. (2011) [42]	Ceftiofur	β-Lactam antibiotic	+	19.1 µmol/L	1.7–41 (highly dependant on T and dilution ratio with water)	nd	Cattle (spiked, “water from farm”)	1.1 %
Loke et al. (2003) [45]	Oxytetracycline	Tetracycline	+	2 and 30 mg/L	nd	nd	Pig (spiked, tank)	nd
Loke et al. (2000) [44]	Tylosin A	Macrolide	+	5 mg/L	<2 days	nd	Pig (spiked, tank)	nd
Mitchell et al. (2013) [49]	Ampicillin, florfenicol, sulfadimidine, tylosin	β-Lactam antibiotic, amphenicol, sulfonamide, macrolide	+	Each 0.001–1.0 mM/L	nd	nd	Cattle (spiked, mixed excrements)	3–6 %
Mohring et al. (2009) [50]	8 Sulfonamides	Sulfonamide	+	2–14 mg/kg	nd	nd	Pig (spiked, biogas plant)	15.2 %
Riemenschneider et al. (2014) [57]	Colistin	Polypeptide antibiotic	−	1, 2, 5, 500 mg/kg	nd	nd	Pig and cattle (spiked, biogas plant)	3–4 %
Schlüsener et al. (2006) [59]	Erythromycin, roxithromycin, salinomycin, tiamulin	Macrolide, ionophore, pleuromutilin	+	2 mg/kg	6–>180 days	nd	Pig (spiked, tank)	nd
Shelver and Varel (2012) [61]	Chlortetracycline	Tetracycline	+	>100 and >300 ng/L (only given in figures)	>21 days at 22 °C, <5 days at 38 and 55 °C	nd	Pig (medicated, mixed excrements)	nd
Shi et al. (2011) [62]	Tetracycline, sulfamethoxydiazine	Tetracycline, sulfonamide	−	Each 25 and 50 mg/L	<12 h	nd	Pig (spiked, mixed excrements)	10 %
Stone et al. (2009) [64]	Chlortetracycline (CTC), tylosin (TYL)	Tetracycline, macrolide	+	28 (CTC), 1.1 (TYL) mg/L	nd	nd	Pig (medicated, manure)	nd
Szatmári et al. (2011) [65]	Doxycycline	Tetracycline	−	61.57 ± 14.26 mg/kg	53 days	nd	Pig (medicated, manure)	nd
Varel (2002) [67]	Carvacrol^a^, thymol^a^	Terpenoid	−	Each 6.7–16.75 mmol/L	nd	nd	Pig (spiked, mixed excrements)	nd
Varel et al. (2012) [68]	Chlortetracycline (CTC), monensin (MON)	Tetracycline, ionophores	−	5.9–8.3 (CTC), 0.3–0.74 (MON) mg/L	nd (DT_50_ not reached for MON)	nd	Pig, cattle (medicated, seed slurry and manure)	4 %
Wang et al. (2014) [72]	Tylosin	Macrolide	−	12 mg/kg	nd	nd	Pig (medicated, spiked, mixed excrements)	nd
Wang et al. (2015) [73]	Oxytetracycline	Tetracycline	+	3746.39 mg/kg	9.04 and 9.65 days	nd	Pig (medicated, spiked, mixed excrements)	22 %
Widyasari-Mehta et al. (2016) [76]	Doxycycline	Tetracycline	+	51 and 20 mg/kg	120 and 91 days	nd	Pig (spiked, tank, biogas plant)	1.8 %
Winckler and Grafe (2001) [78]	Tetracycline	Tetracycline	−	20 and 100 mg/L	55–105 days	nd	Pig (spiked, tank)	nd
Zheng et al. (2012) [80]	17-β-Estradiol, 17-α-estradiol, estrone	Hormone	+	Each 5 mg/L	nd	nd	Cattle (spiked, lagoon water)	nd
Zheng et al. (2013) [81]	17α-Estradiol-3-sulfate	Conjugate of a hormone	+	5 mg/L	23–724 days	nd	Cattle (spiked, lagoon water)	1.2 %

*TP* transformation products considered, *nd* not determined or not defined, *DT*_*50*_ disappearance time 50 %

^a^Biocides

**Table 8:** Studies on the transformation of VMPs and biocides in liquid manure

Author (Year)	Focus and parameters	Setup	Amount of manure	Preconditioning/acclimatization	Replicates	Study-*T* (°C)	Eh (mV)	Study duration (days)
Akyol et al. (2016) [1]	Biogas production, abundance of bacteria (log copy number/100 ng cDNA)	1-L batch digesters continuously stirred	600 mL + 60 mL inoculum from laboratory digester	nd	1 + control	55	nd	20
Ali et al. (2013) [2]	pH and Eh	2.3-L erlenmeyer flask, continuously stirred and flushed with N_2_/O_2_ for different Eh (Fig. 5)	150 g wet lagoon sediment + 1.5 L 0.01 M CaCl_2_	1 week for stabilization of pH and Eh	1	25	(−100), (0), (250), (350)	20
Álvarez et al. (2010) [3]	Biogas composition, pressure, sorption	500-mL glass flasks with coiled butyl rubber stoppers	385 mL + inoculum (granular biomass from an anaerobic internal circulation digester)	Basal medium: cysteine (0.5 g/L), NaHCO_3_ (5 g/L), pH 7.0–7.2; flushing with N_2_, 1.2 mL Na_2_S (20 g/L) (reducing agent)	2	35	nd	21
Angenent et al. (2008) [4]	Antibiotic resistance, methane production, volatile solids removal, VFA	Manure taken from ASBR effluent, 5-mL capped glass serum vials	1 mL	249 days of ASBR operation	1	25	nd	2
Arikan (2008) [5]	Sorption, pH, total solids, volatile solids, total alkalinity, NH_4_-N, COD	1-L batch laboratory digester	800 mL manure + 200 mL inoculum from a dairy manure digester	nd	3	35	nd	33
Arikan et al. (2006) [6]	Biogas production, total solids, total alkalinity, total N, total P	1.225-L batch laboratory digester	1 L manure + 225 mL inoculum from a dairy manure digester	nd	3	35	nd	64
Bailey et al. (2016) [9]	liquid–solid distribution (Kd)	15-mL polypropylene centrifuge tubes	3.3–10 g	14 days at 23 °C	3	23	nd	28
Blackwell et al. (2005) [11]	Exposure assessment, organic carbon, dry matter, available P and N	Closed bottle test, tightly capped and stored without agitation	200 mL	nd	3	20	nd	40
Cetecioglu et al. (2013) [14]	Synthetic manure, COD, biogas production	ASBR, concentration influent and effluent, sludge	1 L	150 days of ASBR operation	1	35	nd	155
Grote et al. (2004) [21]	Metabolism, transformation	Outdoor realistic conditions with continuous influent of contaminated manure	“Barrels”	nd	1	Outdoor	nd	240 + 210
Harms (2006) [25]	Transformation	nd	nd	nd	nd	−20, 7, RT	nd	112, 224
Heuer et al. (2008) [26]	Bacterial community	nd	nd	nd	1	20	nd	172
Höltge and Kreuzig (2007) [27]	Transformation, NER	300-mL flasks, glass stoppers with inlet and outlet valves, ^14^CO_2_ trap	50 g	7 days	3	20	nd	72
Huang et al. (2014) [29]	Biogas production, N-total, COD, pH	1.3-L laboratory model anaerobic three-neck glass reactor	<1300 mL	15 days until equilibrium of biogas production in reactor	3	20	nd	28
Joy et al. (2014) [32]	Antibiotic resistance genes (ARGs)	100-mL glass amber wide mouth	75 g	Sparged for 5 min with nitrogen	2	37	nd	40
Kolz et al. (2005) [34]	Aerobic vs anaerobic, sorption, pH, total solids, N, TOC, P	Amber glass vials with teflon-lined caps, headspace filled with He	20 mL	“Homogenized stored in glass jars at 4 °C until use”	3	22	(−10) to (−160)	3
Kreuzig (2010) [35]	T, Eh, dry matter, O_2_, N-total, NH_4_-N, TOC, BOD	300-mL flasks, glass stoppers with inlet and outlet valves, ^14^CO_2_ trap	50 g	nd	2	5, 10, 20	(− 80)	30, 100, 177
Kreuzig and Höltge (2005) [38]	Transformation, NER	300-mL flasks, glass stoppers with inlet and outlet valves, ^14^CO_2_ trap	50 g	nd	2	20	nd	102
Kreuzig et al. (2007) [36]	Manure-soil mixtures, transformation, NER	300-mL flasks, glass stoppers with inlet and outlet valves, ^14^CO_2_ trap	50 g	nd	2	20	nd	102
Kreuzig et al. (2010) [37]	Manure-soil mixtures, transformation, NER, biocides	300-mL flasks, glass stoppers with inlet and outlet valves, ^14^CO_2_ trap	50 g	nd	2	20	(<120)	177
Kuchta and Cessna (2009) [39]	Sorption, liquid–solid distribution after centrifugation	20-L stainless steel storage container with clipdown cover	15.5 L	nd	2	20	nd	160
Kühne et al. (2000) [40]	Transformation	Vacuum desiccator (Fig. 6)	1 L	nd	2	RT	nd	8
Lamshöft et al. (2010) [41]	T, Eh, dry mass, pH, BOD, COD, total carbon, conductivity	300-mL flasks with ^14^CO_2_ trap	50 g	“The manure was allowed to attain room temperature”	3	10, 20	(− 280) to (− 329)	150
Li et al. (2011) [42]	TOC, conductivity, pH, P, NH_4_-N, Cl^−^, Br^−^, NO3^−^, Na, K, Ca, Fe, Mg, Al, Si, Cu, Zn	Amber 250-mL bottles with teflon-lined caps served as reactors	<250 mL	nd	3	15, 25, 35, 45	nd	72
Loke et al. (2003) [45]	pH, Eh via indicator, freely dissolved fraction	According to ISO 11734 [30], 1-L bottles; titanium(III)citrate as reducing agent	525.0 mL mineral medium, 50.0 mL manure, 100.0 mL stock solution	<2 weeks storage at 4 °C	4	21	nd	180
Loke et al. (2000) [44]	Transformation, filtered vs non-filtered	According to ISO 11734 [30], volumes × 50, 680 mL	680 mL (water with 6.4 % manure)	1 mm sieved, N_2_ bubbled through manure, stored at 4 and −20 °C before usage	4	20	nd	7
Mitchell et al. (2013) [49]	pH, CH_4_, CO_2_ inhibition, total solids (TS) and VSS	300-mL glass serum bottles fitted with rubber septum, headspace filled with N_2_, inoculum used	200 mL	nd	3	37	nd	40
Mohring et al. (2009) [50]	Biogas production, pH	5-L fermentors (Bigatec, Rheinberg, Germany), German VDI 4630 guideline, DIN 38414 part 8, control experiments in 500-mL flasks	1.89 kg manure, 1.89 L water, 0.42 kg inoculum	nd	2	37	nd	34
Riemenschneider et al. (2014) [57]	Biogas production, batch experiment and continuous feeding experiment	20-L laboratory-scale reactor	15 kg	nd	2	38	nd	44
Schlüsener et al. (2006) [59]	Transformation	Erlenmeyer flasks closed with a fermenting tube	100 g	nd	1	20	nd	180
Shelver and Varel (2012) [61]	pH, transformation	2-L digester flasks	nd	nd	3	22, 38, 55	nd	28
Shi et al. (2011) [62]	Methane production, pH, total solids	1-L digester with gas absorbing bottle and collector bottle (Fig. 7)	1 L (including 100 g dry manure, 100 mL inoculum)	nd	3	25	nd	20
Stone et al. (2009) [64]	CH_4_, CO_2_, volatile fatty acids, pH, Alkalinity, COD, VSS, VFA, hydrogenotrophic methanogens, acetoclastic methanogens	120-mL batch reactors, butyl rubber stoppers, headspace N_2_ purged	50 g	105 days at 4 °C	3	10–20 (gradient)	nd	216
Szatmári et al. (2011) [65]	Transformation	300-mL BOD bottles as used in closed bottle tests; referring to VICH [70]	<300 mL	nd	nd	20	nd	112
Varel (2002) [67]	Odor, total gas, VFA, l-lactate, pH	1-L Erlenmeyer flasks, N_2_-gas, rubber stopper	500 mL (feces, urine, distilled water; 50:35:15)	nd	2	25	nd	62
Varel et al. (2012) [68]	Odor, pH, VFA, aromatic fermentation products, methane, coliforms	2-L Erlenmeyer flasks with rubber stopper	600 mL (1:1 seed slurry and fresh manure)	Establishing “seed slurry” over 2–5 months for stabilization of pH, methane and VFA production	2	22, 38, 55	nd	25, 28
Wang et al. (2014) [72]	Biogas production, Shannon’s diversity index (H’), N-total, COD, pH	1.3-L laboratory model anaerobic three-neck glass reactor	<1300 mL	Until equilibrium of biogas production in reactor	3	20	nd	15
Wang et al. (2015) [73]	Transformation products, total N, abundance of Bacillus cereus	3-L open beaker	1200 g	7 days at 23.2 °C	3	23.2	nd	40
Widyasari-Mehta et al. (2016) [76]	Non-extractable residues, redox potential, pH	300-mL flasks, glass stoppers with inlet and outlet valves, CO_2_ trap	75 g	nd	2	20	(−189) to (−21), (−274)	170
Winckler and Grafe (2001) [78]	T, transformation	500-L tanks	295 L	nd	4	8	–	48
Zheng et al. (2012) [80]	T, transformation	250-mL glass bottles with teflon-lined screw caps, glovebox, Na_2_S, N_2_	<250 mL	1 day preconditioning	3	35	(− 277)	52
Zheng et al. (2013) [81]	T, transformation	250-mL glass bottles with teflon-lined screw caps, glovebox, Na_2_S, N_2_	<250 mL	1 day preconditioning	3	15, 25, 35, 45	nd	65

*nd* not determined or not defined

### Citation map

**Fig. 1:**
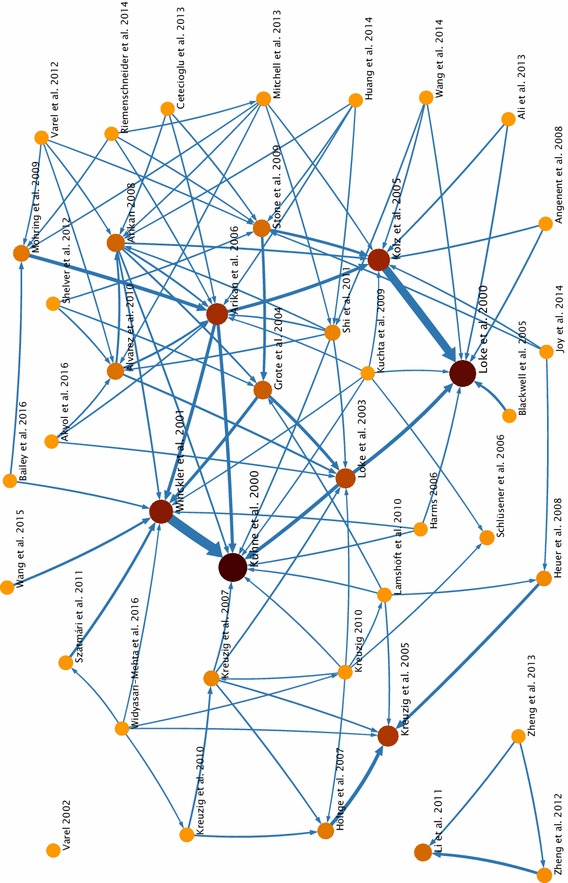
Citation map (generated via http://www.mapequation.org, Edler and Rosvall [17]); each *knot* represents one publication, and each *arrow* visualizes the citation flow

The following citation map (Fig. 1) provides a visualization of the interconnection of the authors/working groups by generating a network and visualizing their respective impact in this field of research. Each node represents one publication. The darker and the bigger the node, the more often the publication is cited. The arrows show who cites whom, and their thicknesses correlate with the citation flow indicating established thematic clusters. Only three publications are completely left out citing each other, owed to dealing with hormones and lagoon water. One isolated work of Varel [67] considers deliberate application of (natural) biocides to manure. This was to stop microbial activity and prevent “odor emissions” during the storage of manure. One cluster is implied on the left of this network, showing all the seven publications, which used ^14^C-labeled compounds, all originating from Germany (working groups Kreuzig and Spiteller). The most often cited publications within the community network of the 42 papers are from Arikan et al. [6], Kolz et al. [34], Kühne et al. [40], Loke et al. [44], and Winckler and Grafe [78]. This is partly explainable by the relatively early dating of these publications.

### Studied substance classes

Equivalent to the application practice in livestock breeding, mainly tetracyclines (20 of 42 studies), sulfonamides (12 of 42 studies), and macrolides (10 of 42 studies) are considered. There are only a few studies with parasiticides. For biocides, only three publications were found [35, 37, 67]. Within 2 of 42 studies, transformation of excreted hormones was investigated. Although they are not about VMPs, these publications are also considered because they are well documented (e.g., measured redox potential) and conducted similar to transformation studies with VMPs.

### Chemical analysis

As already mentioned with regard to the citation map, seven studies used ^14^C-labeled test substances. By this, a mass balance of the experiment considering transformation, mineralization, volatilization, and the formation of non-extractable residues is possible. The methods used are radio thin-layer chromatography (RTLC), oxidizers for solid samples, and liquid scintillation counting (LSC). Only Heuer et al. [26] and Lamshöft et al. [41] further used LC–MS techniques in combination with radio techniques, an approach that will be inevitable in future studies to gain maximum information out of transformation studies in terms of transformation product identification and quantification.

Most of the studies worked with unlabeled substances and used LC–MS/MS for detection and quantification of the VMPs and biocides and their transformation products (24 publications). Some of them combined UV–Vis/diode array detector (DAD) methods with MS methods (4 publications). For example, Schlüsener et al. [59] used HR-MS (high-resolution mass spectrometry) for further salinomycin transformation product identification. Within nine publications, only UV–Vis/DAD detection methods were used. The GC (gas chromatography) method was applied only by Varel [67] for the detection of the terpenoids carvacrol and thymol. Additionally, Varel et al. [68] applied an ELISA method (enzyme-linked immunosorbent assay) for the detection of chlortetracycline.

### Metabolites and transformation products

With regard to VMPs, it is important to distinguish between metabolites, which may be formed in the treated animal, and transformation products, which may be formed from excreted parent compounds and metabolites in the environment.

Transformation products or metabolites were determined in 26 studies. This implies sophisticated methodology by liquid chromatography coupled to preferably tandem mass spectrometry or high-resolution mass spectrometry (LC–MS/MS or LC–HR-MS). For specific applications, HPLC (high-performance liquid chromatography) with UV (ultraviolet) detection may be sufficient [78]. Due to missing reference substances, transformation products are often determined only qualitatively.

For example, Arikan [5] studied in detail the fate of chlortetracycline (CTC) during anaerobic digestion of manure from medicated calves. The CTC concentration decreased about 75 % and the concentration of the CTC epimer, 4-epi-chlortetracycline, declined roughly 33 % during the 33-day experiment. The CTC metabolite iso-chlortetracycline increased twofold in concentration. Referring to a higher water solubility, the authors concluded a possible occurrence of metabolites of CTC in water bodies. Also Mitchell et al. [49] stated that solid and liquid effluents from anaerobic digestion treatment containing antibiotic transformation products could represent an environmental concern.

For example, in the study by Heuer et al. [26], the concentration of sulfadiazine (SDZ) increased by 42 % during the storage of manure due to deacetylation of the metabolite *N*-acetyl-SDZ. Basically, the same was determined by Lamshöft et al. [41] who state that environmental effects may be underestimated, if the parent compound alone was considered for the environmental risk assessment.

### Source of manure

There are different approaches on the application of the test substance to manure in respect of transformation studies. Contaminated manure can be obtained by sampling a tank containing the manure from previously medicated animals. If metabolites are of concern, a more realistic scenario can thus be studied. The deacetylation of the metabolite *N*-acetyl-sulfadiazine in manure, after excretion back to the parent compound sulfadiazine, is a well-studied example [26, 41]. Further, VMPs influence the microbial community structure and thus its own transformation fate in manure. The same applies to the effect of biocides on microbial community. Considering analytical method development, using medicated manure makes it difficult if not impossible to determine the recovery rates of the analytes out of the excreted and then aged manure. At this point, only radioactive methods can provide a valid survey on parent compound excretion and distribution. In the literature under study, only Heuer et al. [26] and Lamshöft et al. [41] worked with radioactive labeled VMPs and medicated manure (^14^C-sulfadiazine, ^14^C-difloxacin). Overall, 16 out of 42 studies were conducted with medicated manure. Additionally, three studies worked with both medicated and spiked manure [29, 72, 73].

In general, spiking manure in laboratory scale is a much more reproducible way of generating contaminated manure and the only way to conduct transformation studies of biocides. By this approach, it is possible to determine recovery rates with unlabeled compounds and to study sorption processes. Nevertheless, Huang et al. [29] report that methane production of manure spiked with chlortetracycline (0.55 mg/kg dw) was reduced by 12 % compared to manure of treated animals with the same chlortetracycline concentration. Further, Wang et al. [72] found a lower diversity index of methanogenic archaea in manure of animals treated with tylosin compared to manure spiked with tylosin at the same concentration. Wang et al. [73] also found differences between spiked manure and manure from treated animals in terms of the abundance of oxytetracycline-degrading *Bacillus cereus* and transformation products of oxytetracycline.

Generally, liquid manure is an anaerobic liquid medium. Samplings taken directly from a tank at a farm represent the most reliable source of liquid manure. By this approach, a microbial community, typical for authentic storage tanks, is used in the transformation experiment, which is not the case if excrements are sampled from diverse animals and mixed afterwards in order to obtain a manure-like medium. Ten out of 42 studies worked with liquid manure taken out of a bigger tank at a farm. In contrast to this, 22 publications report a procedure of mixing more or less fresh excrements with water and in some cases with an inoculum to produce liquid manure on a laboratory scale. Out of these 22, only Varel et al. [68] describe a well-documented procedure of generating a “seed manure” over a time period of 2–5 months to then mix it with fresh manure in order to preserve a reproducible artificial liquid manure.

Four studies worked with lagoon water, which mainly differs from liquid manure in its lower dry matter content of 1.2–3.6 %. Additionally, Li et al. [42] used “recycled water derived from a beef farm.” Within one publication, lagoon sediment was mixed with water down to a dry matter content of 2.7 % [2]. Cetecioglu et al. [14] and Angenent et al. [4] took manure for transformation experiments out of a continuously running anaerobic sequencing batch reactor (ASBR), whereas Mohring et al. [50] and Riemenschneider et al. [57] took it directly out of a biogas plant.

### Matrix characteristics and sorption to suspended solids

From the 42 studies under investigation, 10 used cattle manure featuring dry matter contents from 1.1 up to 15 %. Three studies used both pig and cattle manure. One study relied on a synthetic matrix–water mixture—including volatile fatty acids, glucose, and starch—to approximate properties of liquid manure [14]. Within the remaining 28 studies, pig manure with dry matter contents from 2 up to 22 % was used. Comparing the results is complicated due to the differing dry matter contents. Kreuzig [35] emphasizes substance-specific interactions with the different pig or cattle manure matrices. He further mentions that the dry substance content of manure can be one of the most relevant factors affecting the transformation of VMPs and biocides. In a study on the stability of tylosin A in manure, Loke et al. [44] could not clarify whether the decrease in the concentration of this compound is caused by sorption or abiotic or biotic chemical degradation. Similarly, Shi et al. [62] could not explain whether the rapid disappearance of the investigated antibiotics tetracycline and sulfamethoxydiazine could be due to their adsorption onto solid materials or degradation by microorganisms. In another study, Loke et al. [45] stated that very low free concentrations of oxytetracycline and metabolites in an anaerobic degradation experiment are due to the high amounts of substances being bound to particles in the manure matrix rather than to the degradation to unknown compounds.

In 17 studies, this dry matter content, which is strongly influencing sorption of the test substances, is not even mentioned and thus prohibiting a deeper interpretation of the results. Dry matter content is a key parameter, which impacts the dissipation rates, as shown by Álvarez et al. [3], Arikan [5], Kolz et al. [34], Kreuzig [35], and Kuchta et al. [39]. These authors investigated explicitly sorption onto solid matter, which had already been recognized as a crucial parameter by Winckler and Grafe [78].

### Experimental setup

A wide variety of experimental setups were used in the different studies analyzed. The amount of manure used for one replicate ranges from 1 mL [4] up to 295 L [78]. By far, most of the studies were conducted with 50–500 mL manure. Ten studies do not report a clearly defined amount of manure used. Most studies seem to have been conducted without any agitation of the manure during the experiments or they do not clearly report it. There are only a few studies, which mention a periodical stirring of the test manure or at least a stirring directly before sampling the manure.

Some studies refer to several guidelines. Loke et al. [44, 45] refer to ISO 11734 [30], Mohring et al. [50] refer to the German VDI 4630 [69] guideline and to DIN 38414 part 8 [16], and Szatmári et al. [65] refer to the former draft of the EMA guideline on determining the fate of veterinary medicinal products in manure [19, 70].

Many of the studies try to establish anaerobic conditions using an inert gas for flushing headspace or solutions at the beginning of the experiments. Flow-through systems and batch systems (static or semi-static systems) are used. There is only one publication reporting a real flow-through system [2]. Ali et al. worked with a continuous flow-through of N_2_ and O_2_ in a defined ratio to establish a redox potential between −100 and +350 mV. With the addition of HCl or NaOH, the pH was adjusted.

Nearly all of the other studies report the usage of batch systems. They just use an inert gas at the beginning of the experiment (static) or periodically during the experiment (semi-static) to guarantee anoxic conditions for the sampling or to drive out the generated biogas. Nevertheless, it is often not well described how exactly produced biogas was driven out of the system or how it was dealt with the generated biogas overpressure. This is important for studies monitoring biogas production or for those studies working with ^14^C and monitoring mineralization, by trapping ^14^CO_2_ with a KOH solution for example [35]. Arikan et al. [6] used a water displacement technique to monitor biogas production. Mitchell et al. [49] worked with a syringe methodology to measure biogas production.

Others—not measuring biogas production—worked with closed glass bottles [11]. Kolz et al. [34] used amber glass vials with Teflon-lined caps. To achieve similar storage conditions as given during storage under a plastic cover in lagoons, Kuchta et al. [39] conducted their studies in 20-L stainless steel containers with clipdown covers. Schlüsener et al. [59] used 300-mL Erlenmeyer flasks closed with fermenting tubes. Mohring et al. [50] worked with a commercially available anaerobic 5-L fermentor (Bigatec, Rheinberg, Germany). Winckler and Grafe [78] were one of the first to study the transformation of pharmaceuticals in liquid manure. They worked with very large 500-L tanks under outdoor and temperature-controlled conditions. In any case, these examples illustrate the aforementioned huge variety of different experimental approaches.

In contrast to the batch experiments, there are two publications working with an anaerobic sequencing batch reactor (ASBR). In the study of Angenent et al. [4], a 5-L ASBR was run by sequencing through a feed step, a react step, a settling step, and a decant step. An intermittent mixing process was thereby performed by biogas recycling. Tylosin half-life experiments were conducted by taking manure from the ASBR, placing it into capped 5-mL glass serum vials (pre-purged with N_2_), and spiking with tylosin. The vials were stored for a duration of 48 h at a temperature of 25 °C in a water bath. Cetecioglu et al. [14] also used an ASBR with a 24-h cycle to measure the impact of tetracycline on biogas production and the biodegradation of a synthetic organic substrate. In contrast to Angenent et al. [4], they monitored the tetracycline mass balance between the influent and the effluent of the ASBR considering the sludge inside the ASBR. With this setup, it was not possible to determine DT_50_ values. Huang et al. [29] and Wang et al. [72] also worked with a kind of ASBR. Riemenschneider et al. [57] worked with both a batch experiment and a constant feeding experiment using a 20-L laboratory-scale reactor. Grote et al. [21] only conducted the transformation study with a realistic outdoor scenario, working with chlortetracycline (CTC), sulfadiazine (SDZ), and trimethoprim (TMP). The medicated pigs excreted the pharmaceuticals over a long period of time, thus all relevant metabolites were considered. It is not easily possible to determine DT_50_ values for this approach, but it is possible to study the realistic transformation of a pharmaceutical.

### Parameters

Summarizing, all the studies measured or controlled at least some of the following physical, chemical, and biological parameters during the transformation studies: redox potential Eh (mV), dry matter content (%), pH, dissolved O_2_ content (mg/kg), NH_4_-N (g/kg), *N*_total_ (g/kg), total organic carbon (TOC) (g/kg), total carbon (g/kg), biological oxygen demand (BOD) (g/kg), chemical oxygen demand (COD) (g/kg), temperature (°C), volatile suspended solids (VSS), conductivity (µs/cm), P, Cl^−^, Br^−^, NO_3_^−^, Na, K, Ca, Fe, Mg, Al, Si, Cu, Zn, relative abundance of hydrogenotrophic methanogens and acetoclastic methanogens, volatile fatty acids (VFA) and aromatic fermentation products (l-lactate, acetate, propionate, isobutyrate, butyrate, isovalerate, valerate, isocaproate, caproate, phenol, *p*-cresol, indole and skatole), methane/biogas production, mineralization (%), 50 and 90 % disappearance time (DT_50_, DT_90_), transformation products (TP), non-extractable residues (NER), mass balance/recovery (%), Shannon’s diversity index (H’), log copy number/100 ng cDNA, liquid–solid distribution (*K*_d_), and the number of antibiotic resistance genes (ARGs).

### Aerobic vs. anaerobic conditions

The attempt of this literature review was to select studies that were conducted under primarily anaerobic conditions. The information is either stated by the author(s) or can be deduced from the given facts on the experimental setup. However, also studies are included that used aerobic conditions (e.g., redox potentials above −100 mV, [52]). Various authors merely assume anaerobic conditions without any further indication. Besides, different authors use different terminologies, such as “anaerobic digestion,” “anaerobic conditions,” “methanogenic conditions,” and “anaerobic tightly capped vessels.” Studies with this vague information were still considered in this examination as otherwise the relevant publications would have been limited to a number of only seven studies reporting a redox potential.

Besides using closed laboratory setups, many studies report the use of N_2_ or He gas to purge the headspaces of the systems or to purge the used liquids and manures before starting the experiments. Others used the glovebox technique [80] or added reducing agents to the manures to guarantee reducing conditions (e.g., Na_2_S by Álvarez et al. [3] or titanium(III)citrate by Loke et al. [45]). Loke et al. [45] further added resazurin as a redox indicator. As the test bottles did not show a reddish coloring, they assumed anaerobic conditions. However, it might be difficult to interpret the coloring of this also pH-dependent indicator in deep brown liquid manure. For this, they also monitored methane gas production as a main indicator for methanogenic and, by this, anaerobic conditions. Varel et al. [68] systematically studied methane production of their seed manure before starting transformation studies with this manure in order to be able to work with stable methanogenic/anaerobic conditions.

Kuhne et al. [40] used closed incubation systems to investigate the stability of tetracycline in pig manure. They determined that the DT_50_ for tetracycline in their unventilated systems was 9 days, whereas it was 4.5 days when the slurry was ventilated.

Szatmári et al. [65] compared an anaerobic laboratory study with a field study using manure composting. In the laboratory experiment, more than 30 % and in the field study about 10 % of the initial doxycycline amount could be detected in manure samples after 12 and 16 weeks of aging, respectively. The half-life of doxycycline in manure was calculated to be 52.5 days under anaerobic conditions and 25.7 days under aerobic conditions.

Ali et al. [2] were the only of all included studies who deliberately varied the redox potential. They established a set of microcosms with controlled redox potentials (Eh) (−100 mV, 0 mV, +250 mV, and +350 mV) and pH conditions (pH 5.5, 7.0, and 8.5). With increasing Eh—that is with increasing aerobic conditions—they found significantly higher dissipation rates for tylosin and could attribute this to microbial activity by comparison with sodium azide-treated samples. Increasing pH resulted in increasing dissipation rates. With the addition of azide, a decrease of Eh could be observed. Kolz et al. [34] worked with redox potentials of slurry between −10 and −80 mV. The addition of azide resulted in a decrease of the redox potential to between −90 and −160 mV.

Although redox potential is not directly a proof of anaerobic conditions, it is relatively easy to measure in liquid manure. The internationally harmonized OECD guideline 308 on the transformation of chemicals in water/sediment systems pragmatically sets a maximum upper limit of −100 mV [52] for anaerobic conditions. Strictly considering this limit of −100 mV, only 3–4 studies meet the requirements for anaerobic conditions (Ali et al. [2], Lamshöft et al. [41], Widyasari-Mehta et al. [76], Zheng et al. [81]). It should be considered that redox potentials measured in real manure tanks are even far below −100 mV [74]. Generally, the transformation of VMPs in manure is faster and more complete under aerobic conditions than under anaerobic conditions.

### Methane production and microbial activity

The production of methane was considered as an ongoing parameter in 12 studies. In two studies with tetracyclines, Arikan et al. [6] and Álvarez et al. [3] found that methane production was reduced by 27 % during batch experiments and up to 62 % due to antibiotic dosage, respectively. Stone et al. [64] found that the generation of methane was inhibited by 27.8 % due to the presence of chlortetracycline. Dependent on the dosage, Cetecioglu et al. [14] determined adverse impact of tetracycline with a total collapse of the microbial activity and metabolic functions at a concentration of 8.5 mg/L in a synthetic substrate mixture under anaerobic conditions. Shi et al. [62] found a dosage-dependent inhibition on CH_4_ production and concluded that antibiotics appear to inhibit bacterial activity, resulting in a delay and overall decline in CH_4_ production. Among these 12 studies measuring biogas production, four studies were explicitly concerned with microbiological issues [1, 26, 64, 72]. Varel et al. [68] mention that an adaption period of 5–6 months was necessary for acclimatization of microorganisms to monensin and to reduce effects of antimicrobials on methane production. Others added small amounts of an inoculum from an anaerobic digester at a farm, or from a wastewater treatment plant, or a biogas plant [3, 5, 49, 50, 62]. Besides the topic of transformation in manure, Ma et al. [47] for example isolated a novel strain of bacteria capable of degrading 100 % tylosin within 72 h. Against that background, each future transformation study has to be analyzed critically. Composition of microbial community has a massive effect on transformation rates and routes. Without any further qualitative and quantitative critical analysis of microbiology, it is not possible to produce reliable and reproducible transformation data of VMPs and biocides in liquid manure. From a regulatory point of view, this topic could enable a massive manipulation of transformation data. Therefore, using a reference substance for transformation studies seems necessary.

### Biotic vs. abiotic transformation

In some studies, either sterilization with sodium azide or autoclaving allowed to differentiate between abiotic and biotic transformation. Generally, biotic transformation rates can be substantially higher than pure abiotic transformation rates. However, the process of formation of non-extractable residues cannot be elucidated by this approach.

Loke et al. [45] worked with autoclaved and non-autoclaved manure to study the transformation of oxytetracycline (OTC) by measuring the free concentration of the VMP. By this, they did not find a difference between the sterile and the non-sterile setups, due to the fast sorption of OTC to the solid phase. They did not address the question of whether the following biotic transformation is inhibited by sorption of the substance to suspended solids.

Loke et al. [44] studied the transformation of tylosin A but were unfortunately not able to figure out whether the rapid decrease in the concentration of tylosin A is caused by sorption or abiotic or biotic chemical degradation. Ali et al. [2] later reported, among other conclusions, that microbial degradation was much greater under aerobic (Eh +350 mV) conditions than under anaerobic conditions as compared to abiotic transformation or sorption. Kolz et al. [34] concluded that both biodegradation and abiotic degradation occur during the transformation of tylosin. However, strong sorption to slurry solids was probably the primary mechanism of tylosin disappearance.

Zheng et al. [80] found that the transformation of 17α-estradiol, 17β-estradiol, and estrone was mainly dominated by biodegradation rather than by physical or chemical transformation.

Li et al. [42] found that the combined processes of hydrolysis and biodegradation were responsible for the transformation of ceftiofur. The determined hydrolysis and total degradation rate constants in aqueous solutions varied according to temperature.

### Study temperature

Study temperatures within the considered publications ranged from 5 to 55 °C. Only few publications explicitly analyzed the effects caused by a variation of the study temperature.

Harms [25] examined the stability of pharmaceuticals in manure during storage at 7 °C and found no degradation of chlortetracycline during 6 months. Sulfadiazine was reduced to 50 % after 1 week, but the remaining residuals were stable until the end of the trial (32 weeks). The author varied the storage temperature (−20, 7 °C, room temperature) over a period of 16 weeks. Sulfamerazine, sulfamethoxypyrazine, sulfaguanidine, and sulfisomedine persisted. Sulfamethoxazole was reduced up to 80 % at 7 °C as well as at room temperature. The same degradation rate was reached more quickly with higher temperatures than with cooler conditions of storage. Enrofloxacin and tiamulin were reduced to 20 % at 7 °C storage temperature. For tiamulin, a reduction of 10 % at 7 °C and 25 % at room temperature was observed. Only sulfapyridazine and enrofloxacin showed a small decline at a storage temperature of −20 °C.

Li et al. [42] studied the transformation of ceftiofur at temperatures between 15 and 45 °C and found increasing hydrolysis and biodegradation rates with temperature. Increasing the study temperature from 35 to 45 °C resulted in an increasing relevance of hydrolysis on transformation of ceftiofur, whereas biodegradation remained static.

Similarly, Varel et al. [68] found principally increasing dissipation rates with increasing the temperature from 22 to 55 °C. They studied the effect of anaerobic digestion at different temperatures, among other parameters, on the fate of chlortetracycline (CTC) in pig manure and monensin (MON) in cattle manure. The authors concluded that anaerobic digestion at elevated temperatures may be an effective treatment to reduce CTC but not MON. Transformation of CTC mainly depends on abiotic transformation. This was also shown by Shelver and Varel [61] who also worked with CTC between 22 and 55 °C.

Stone et al. [64] worked with a temperature gradient to simulate field conditions commonly found in the northern mid-western United States of America. They started with 10 °C (0–30 days) and increased the temperature over time: 12 °C (30–46 days), 15 °C (46–56 days), and 20 °C (56–216 days).

Half of the studies (21 out of 42) were conducted at ambient temperatures (ranging from 20 to 25 °C). Some other experiments were performed at elevated temperatures of 35 to 40 °C, which enhances the microbial activity [5, 32, 57]. In general, transformation was found to be dependent on the study temperature; increasing temperatures simultaneously result in increasing transformation rates. Working with temperatures above the microbiological relevant limit of 35–40 °C results in an inhibition of microbial activity and biodegradation processes. In which way this affects transformation processes mainly depends on the transformation routes of different substances.

## Conclusions

The available studies on the transformation of biocides and veterinary medicinal products in manure show large variations in the experimental setup and conditions such as temperature, redox potential, matrix effects, and physico-chemical properties.

Most frequently investigated VMPs belong to the class of antibiotics, namely sulfonamides, tetracyclines, and macrolide antibiotics. In 16 out of the 42 studies, excrements or manure from a medicated livestock was considered; all other studies are based on spiked manure. There were only three publications working with biocides, which clearly shows that this topic is largely neglected by research. Most of the studies considered manure originating from pigs (28 out of 42), while the remaining studies investigated manure from cattle or in one case synthetic manure was used. Study duration and temperature ranged from 2 to 374 days and 5 to 55 °C, respectively. Many studies do not report the basic parameters. Only seven publications give information on the redox potential of the manure used for transformation. Further, the characterization of the matrix in many cases is inadequate due to missing basic parameters such as dry matter content and pH. Information on dissipation rates or half-lives, transformation products, formation of methane, microbiology, and non-extractable residues (NER) are not available in the majority of the considered publications.

Overall, it can be stated that the majority of the studies describe at least one fundamental parameter of the experimental conditions poorly. Considering all the different approaches, one can conclude that it is inevitable to give specific guidance for studies on the transformation in manure in general and especially with regard to the applicability and acceptability of studies in regulatory contexts. All the parameters—as they are studied particularly within single publications—affect the basic outcome of a transformation study. It is therefore necessary to standardize them or at least measure and report them individually. Parameters such as temperature, dry matter content, origin and preconditioning of the manure, microbiology, pH, and redox potential do have massive effects on the transformation rates or routes of VMPs and biocides in liquid manure. Some of these parameters are relatively simple to measure and should be monitored mandatorily, or even standardized where possible. Only one transformation study at real manure storage tanks was found. This is an important area for research to study transformation processes under realistic conditions. Comparing such authentic outdoor results with those produced on laboratory scale is important to validate the outcome of laboratory studies and to evaluate different experimental setups. One of the most challenging topics with knowledge gaps is related to the composition, the development or spread of resistance, or the adaption and activity of the microbial community. For future studies, this topic should always be addressed.

As an outcome of the summarized monitoring studies, it can be concluded that VMPs occur ubiquitously in manure. Biocides are totally neglected until today. For sulfadiazine and chlortetracycline, concentrations exceeding predicted environmental concentrations were found. For future monitoring studies, it should be obligatory in terms of environmental risk assessment to define the exact animal type as the origin of the manure and to measure and report nitrogen content and dry matter content of the analyzed manures. From our point of view, it would be helpful to imply a Europe-wide monitoring program to evaluate the calculated PECs of VMPs in liquid manure.

## Abbreviations

ASBR: anaerobic sequencing batch reactor
BOD: biological oxygen demand
CAFO: concentrated animal feeding operation
COD: chemical oxygen demand
CTC: chlortetracycline
CYN: cyanamide
DAD: diode array detector
DIF: difloxacin
DIN: German Institute for Standardization (german: Deutsches Institut für Normung e.V.)
DT_50_: time needed for disappearance of 50 % of the parent compound, disappearance time
DT_90_: time needed for disappearance of 90 % of the parent compound, disappearance time
dw: dry weight
ELISA: enzyme-linked immunosorbent assay
EMA: European Medicines Agency
ERA: environmental risk assessment
ERY: erythromycin
EU: European Union
F + E: research and development (German: Forschung und Entwicklung)
FEN: fenbendazole
FKZ: project no. (German: Forschungskennzahl)
FLU: flubendazole
GC: gas chromatography
HPLC: high-performance liquid chromatography
HR-MS: high-resolution mass spectrometry
IMZ: imazalil
ISO: International Organization for Standardization
LC: liquid chromatography
LIN: lincomycin
LOD: limit of detection
LSC: liquid scintillation counting
MEC: measured environmental concentration
MON: monensin
MS: mass spectrometry
nd: not determined or not defined
NER: non-extractable residues
ns: not specified
OECD: Organisation for Economic co-operation and Development
OTC: oxytetracycline
PEC: predicted environmental concentration
RTLC: radio thin-layer chromatography
SCP: sulfachloropyridazine
SDZ: sulfadiazine
SMZ: sulfamethoxazole
SPN: spectinomycin
TMP: trimethoprim
TOC: total organic carbon
TP: transformation product
TS: total solids
TYL: tylosin
UV: ultraviolet
VDI: Association of German Engineers (German: Verein Deutscher Ingenieure)
VFA: volatile fatty acid
VICH: Veterinary International Conference on Harmonization
VMP: veterinary medicinal product
VSS: volatile suspended solids
ww: wet weight
